# Seed Bio-priming of wheat with a novel bacterial strain to modulate drought stress in Daegu, South Korea

**DOI:** 10.3389/fpls.2023.1118941

**Published:** 2023-04-27

**Authors:** Shifa Shaffique, Muhammad Imran, Sang-Mo Kang, Muhammad Aaqil Khan, Sajjad Asaf, Won-Chan Kim, In-Jung Lee

**Affiliations:** ^1^ Department of Applied Biosciences, Kyungpook National University, Daegu, Republic of Korea; ^2^ Biosafety Division, National Institute of Agriculture Science, Rural Development Administration, Jeonju, Republic of Korea; ^3^ Department of Chemical and Life Sciences, Qurtuba University of Science and Information Technology, Peshawar, Pakistan; ^4^ Natural and Medical Sciences Research Center, University of Nizwa, Nizwa, Oman

**Keywords:** germination, seed biopriming, newly isolated strain, wheat, drought

## Abstract

Wheat is one of the major cereal crop grown food worldwide and, therefore, plays has a key role in alleviating the global hunger crisis. The effects of drought stress can reduces crop yields by up to 50% globally. The use of drought-tolerant bacteria for biopriming can improve crop yields by countering the negative effects of drought stress on crop plants. Seed biopriming can reinforce the cellular defense responses to stresses via the stress memory mechanism, that its activates the antioxidant system and induces phytohormone production. In the present study, bacterial strains were isolated from rhizospheric soil taken from around the Artemisia plant at Pohang Beach, located near Daegu, in the South Korea Republic of Korea. Seventy-three isolates were screened for their growth-promoting attributes and biochemical characteristics. Among them, the bacterial strain SH-8 was selected preferred based on its plant growth-promoting bacterial traits, which are as follows: abscisic acid (ABA) concentration = 1.08 ± 0.05 ng/mL, phosphate-solubilizing index = 4.14 ± 0.30, and sucrose production = 0.61 ± 0.13 mg/mL. The novel strain SH-8 demonstrated high tolerance oxidative stress. The antioxidant analysis also showed that SH-8 contained significantly higher levels of catalase (CAT), superoxide dismutase (SOD), and ascorbic peroxidase (APX). The present study also quantified and determined the effects of biopriming wheat (Triticum aestivum) seeds with the novel strain SH-8. SH-8 was highly effective in enhancing the drought tolerance of bioprimed seeds; their drought tolerance and germination potential (GP) were increased by up to 20% and 60%, respectively, compared with those in the control group. The lowest level of impact caused by drought stress and the highest germination potential, seed vigor index (SVI), and germination energy (GE) (90%, 2160, and 80%, respectively), were recorded for seeds bioprimed with with SH-8. These results show that SH-8 enhances drought stress tolerance by up to 20%. Our study suggests that the novel rhizospheric bacterium SH-8 (gene accession number OM535901) is a valuable biostimulant that improves drought stress tolerance in wheat plants and has the potential to be used as a biofertilizer under drought conditions.

## Graphical Abstract

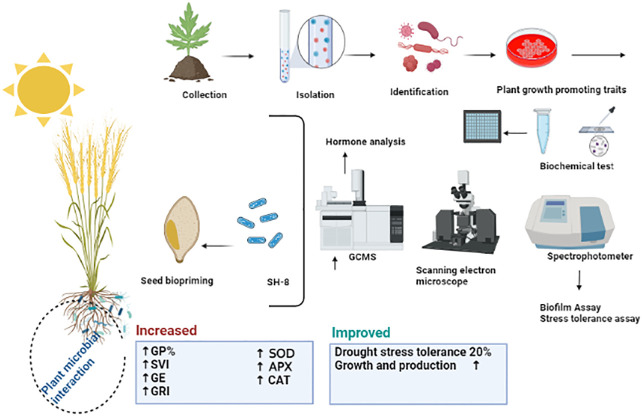

## 1 Introduction

Plants are sessile in nature and are frequently exposed to unfavorable circumstances, such as biotic and abiotic stresses, which negatively affect their yield and survival (Rhodes and Nadolska-Orczyk, 2001; Shabala, 2017). Abiotic stress results in the reduction of crop yields by approximately 70% worldwide. It has been shown that, among the various environmental stresses that limit grain production, drought stress is the most damaging (Arzanesh et al., 2011; Zhu, 2016; Francini and Sebastiani, 2019); according to published statistics, it causes an approximate reduction of 50% in crop productivity (Boken et al., 2005; Sallam et al., 2019).

Drought is water scarcity stress caused by a reduction in or lack of water availability. Plant exposure to severe water stress can cause metabolic impairment and restrict photosynthesis, as water molecules act as electron donors in the biochemical process of photosynthesis. It is noteworthy that 35%–45% of the earth is semiarid, that is, characterized by a scarcity of water that is due to a lack of water sources or reduced rainfall (Farooq et al., 2012; Rahdari and Hoseini, 2012). A drought is an unpredictable form of stress because it can be due to either reduced rainfall or a lack of water resource availability (Siddique et al., 2000; Shanker et al., 2014). Climate change and population growth have resulted in droughts being more frequent and severe than in the early 2000s (Farooq et al., 2009; Da Silva et al., 2011; Basu et al., 2016). When imposed before the vegetative stage, drought stress results in the loss of entire crop yields, and when imposed during the vegetative phase, it results in moderate damage to crops. Crops are most susceptible to damage from water deficits during the pollen meiosis phase (Ahanger et al., 2016; Anjum et al., 2017; Ozturk et al., 2021).

Drought stress negatively affects the germination index, energy distribution, potential, and seed vigor index of plants. Seed hydration is essential for the activation of the enzymes that are responsible for seed germination. Drought stress reduces the activity of germinating enzymes, and thus the germination potential of seeds. It has been shown that conventional strategies for enhancing drought tolerance have been shown to have limited effectiveness in the mitigation of the effects of such stress. Therefore, the introduction of new techniques that could help alleviate the effects of drought stress is urgently needed (Liu et al., 2015; Wahab et al., 2022).

Beneficial bacteria form a symbiotic relationship with their host plant: they interact with plants and promote the production of chemical metabolites, phytohormones, enzymes, and the expression of stress-related genes that mitigate the effects of various stresses (Khan et al., 2017a; Shah et al., 2021; Tariq et al., 2021). These beneficial bacteria also induce systemic resistance (ISR) (Yasin et al., 2018). Scientists have recommended the use of beneficial microorganisms as a promising approach to mitigating drought stress (Khan et al., 2017b). It has also been shown that the use of microbes as biostimulants could potentially play a vital role in mitigating drought stress in various crops (Creus et al., 2004; Arzanesh et al., 2011; Kasim et al., 2013; Gontia-Mishra et al., 2020; Kour et al., 2020; Shaffique et al., 2022b). Plant–microbe interactions that assist in agricultural production hold immense potential to address some of the key current global food security challenges (Tariq et al., 2021). Seed biopriming is a pre-germination enhancement procedure in which seeds are inoculated with beneficial microorganisms (Reddy, 2012; Mahmood et al., 2016; Prasad et al., 2016; Singh, 2016) and has been shown to increase drought tolerance in crops. Seed germination is the process by which seedlings sprout from plant seeds, and its success is critical to plant survival. Various parameters can affect germination, such as humidity, pH, temperature, and light (Nonogaki et al., 2010; Bewley and Black, 2013; Yan, 2015). However, germination can be enhanced through various techniques, such as biopriming, chemo-priming, and hydropriming (Thomas, 1981; Mahmood et al., 2016; Zhang et al., 2019).

Previously, agrochemicals were used to improve crop productivity and stress tolerance; however, rising concerns related to the exotoxic behavior of agrochemicals, and their effects on all components of the ecosystem have led to minimizing their application and promoting the use of biostimulants with minimal side effects (Lamberth et al., 2013; Saravanan et al., 2022). Seed biopriming is a recently adopted method that allows microorganisms to adhere to and acclimate to seeds. It ensures germination and enhances seed vigor index and early seedling characteristics. It is also a useful tool for alleviating ecological stress in plants (Mitra et al., 2021; Nawaz et al., 2021). Seed biopriming is therefore a promising approach that can be used to attain sustainable agriculture production. It is also environmentally friendly and effective in improving plant yield under extreme ecophysiological conditions (Kumari et al., 2018; Kumari et al., 2021).

Various recent studies have demonstrated the significant effect of seed biopriming against drought stress (Negi and Bharat, 2021; Fatih et al., 2022). It has been found that biopriming up-regulates stress genes (i.e., *DREB, WRKY, EDR, LEA, GolS, NCED*, and *CYP707A*), stimulates plant growth by promoting the production of phytohormones, such as abscisic acid (ABA), jasmonate, salicylic acid, indole acetic acid (IAA), and brassinosteroids, and provides biological control through the production of organic compounds (Athira et al., 2022; Chin et al., 2022). Various studies on different crops suggest the importance of seed biopriming with several bacterial strains (Mahmood et al., 2016; Rajendra Prasad et al., 2016; Song et al., 2017). The application of seed biopriming enhances drought tolerance in various widely grown crops, such as rice (Bashyal et al., 2020; Bashyal et al., 2021), tomato (Anbazhagan et al.; Pehlivan et al., 2018), wheat (Gangwar and Singh, 2018), and alfalfa (Lahrizi et al., 2021). The present study was conducted in wheat seedlings because wheat is the most important nutrient-rich cereal crop, a factor that has markedly increased global demand for this crop. It is, after rice, the most commonly cultivated staple crop worldwide. The global production of wheat is 760 million tons. To meet the increasing demand of the expanding global population, wheat production needs to increase, especially in areas subject to environmental stresses such as drought stress (Vurukonda et al., 2016; Kour et al., 2019a).

The aim of the current study was to screen a range of bacterial isolates to assess their bioefficacy in mitigating the effects of drought stress in wheat plants. It was hypothesized that the rhizospheric soil around the *Artemisia* plant may contain a greater number of microbes that undergo osmotic modulation and could therefore be effective in improving wheat crop productivity by alleviating the effects of drought stress. Therefore, as a pilot study, we investigate their ability to alleviate the effects of drought stress in wheat plants through seed biopriming. In the present study, we evaluate the plant growth-promoting characteristics of new bacterial isolates. We also report their role in enhancing the germination metrics in wheat plants.

## 2 Experimental methodology

### 2.1 Isolation of the bacterial strain SH-8

The bacterial strains were isolated from the rhizosphere soil of the *Artemisia vulgaris* plant at Pohang Beach, in the Republic of Korea. The rhizospheric plant root samples were packed in sterilized polythene airtight bags and kept in an ice box for safe transportation to the Crop Physiology Laboratory in the Department of Plant Biosciences, Kyungpook National University, Daegu, Republic of Korea. The rhizospheric soil (1 g), which was collected from plant roots, was serially diluted (10^−1^ to 10^−9^) using 9 mL of sterile saline solution (0.85%). The bacteria were isolated through inoculation on Luria broth (LB) agar media plates. The plates were incubated at 28−30°C until the appearance of bacterial populations. The pure bacterial colonies were purified by restreaking on LB agar media plates. After a 24-h incubation, the obtained pure colonies of bacterial strains to be investigated further were identified based on their gross morphological features.

### 2.2 Selection of bacterial strains

Bacteria were selected by characterizing their growth-promoting attributes, such as their phosphate solubilization potential, siderophore production, exopolysaccharides, and IAA production. Based on the best plant growth-promoting (PGP) traits, the SH-8 strain was selected and screened with different concentrations (i.e., 5%, 10%, 15%, 20%, and 25%) of polyethylene glycol (PEG).

### 2.3 Screening for phosphate solubilization

The phosphate solubilization of the novel bacterial strain was analyzed in accordance with the procedure described by Khan et al. (Khan et al., 2021), with slight modifications (Wan et al., 2020; Khan et al., 2021). The pure bacterial culture (1 µL) was grown on trypticase soy agar medium supplemented with tricalcium phosphate [Ca_3_(PO_4_)_2_] and incubated at 25−30°C for 7 days. To assess phosphate solubilization capacity, we regularly examined the plates for the development of transparent halos around the bacterial population, as these are an indicator of phosphorus solubilization potential. The phosphate solubilization index (PSI) was determined by measuring the area of the transparent halos around an isolated growing colony.

The PSI was calculated by taking the ratio of the total diameter (i.e., colony + halo zone) to the colony diameter, as described by Rashid et al. and Karpagam and Nagalakshmi et al. (Rashid et al., 2004; Karpagam and Nagalakshmi, 2014).

The equation is given as follows:


(1) PSI = total diameter/colony diameter

### 2.4 Screening for siderophore production

To determine if siderophores were produced, we carried out an agar diffusion assay: 2 μL of the pure bacterial culture was inoculated onto agar plates containing Chrome Azurol S (CAS) and siderophore production was assessed from the size of the orange haloes that developed around the bacterial colonies. The plates were sealed with parafilm and incubated at 25−30°C for 72 h (Arora et al., 2001; Kang et al., 2018).

### 2.5 Screening for IAA production

Equal amounts of pure bacterial isolates were mixed with Salkowski’s reagent (50 mL of 35% HClO_4_, 1 mL of 0.5 M FeCl_3_), and the mixture was placed in the dark for 0.5 h. A change of color from white to pink indicated the presence of IAA (Thuler et al., 2003; Etesami et al., 2015).

### 2.6 Microbial screening while using polyethylene glycol

The drought tolerance of the bacterial isolate was quantified by using a polyethylene glycol (PEG) 6000 assay, which induces artificial osmotic stress. The microbial strain was screened against PEG. Various concentrations of PEG 6000 (i.e., 0%, 5%, 10%, 15%, 20%, 25%, and 30%) were prepared and autoclaved for sterilization. Test tubes, each containing 5 mL of one concentration of sterilized PEG, were inoculated with the bacterial isolate SH-8 (0.1%) and put into a shaking incubator for 24 h at 25–30°C. A UV spectrophotometer (PG Instruments Ltd., UK) was used to measure the optical density at 600 nm. The procedure followed was that described by Asaf et al. and Kim et al. (Asaf et al., 2017; Kim et al., 2020).

### 2.7 Identification and phylogenetic analysis

Genomic DNA (gDNA) was extracted from the SH-8 strain using a DNeasy plant mini kit (Qiagen, Valencia, CA, USA). The 16S rRNA gene was amplified by PCR, sequenced, and identified using the BLAST algorithm by comparison with sequences in the NCBI database. The universal primer pairs 5′-AGAGTTTGATCACTGGCTCAG-3′ and 1492R (5′-CGGCTTACCTTGTTACGACTT-3′) were used to amplify the 16S rRNA sequence. We utilized the MEGA X (version 7.222) CLUSTAL-W program to align highly similar sequences (Tamura et al., 2013). The phylogenetic tree was constructed using the maximum likelihood (ML) technique embedded in MEGA X. Bootstrap replications (1,000) were used to provide statistical support for each node in the phylogenetic trees. The sequence data were deposited in gene bank accessions [*Klebsiella aerogenes* strain SH-8 (OM535901; https://www.ncbi.nlm.nih.gov/nuccore/OM535901.1?report=GenBank)].

### 2.8 In vitro biopriming experiments

A fully factorial design experiment was carried out in a complete randomized block design with five replications. The entire protocol was followed as previously described (Fathollahy and Mozaffari, 2020), with slight modifications. The annual wheat seeds and the novel bacterial strain SH-8 (gene accession number OM535901) were obtained from the Crop Physiology Laboratory, Kyungpook National University, Daegu, Republic of Korea. The entire experimental procedure was conducted on a clean bench with high-efficiency particulate air (HEPA) filters to prevent contamination and maintain a higher level of air purity. Seeds were sterilized with 0.1% Bavistin for 30 minutes and 0.5% sodium hypochlorite for 3 minutes and then rinsed three times with sterile distilled water. The seeds were divided into eight experimental groups as follows: control, microbial pellet, microbial solution, and microbial pellet plus different concentrations of PEG (i.e., 5%, 10%, 15%, 20%, and 25%). The groups were specifically treated as follows: (a) control, sterilized distilled water only; (b) seed biopriming with a bacterial solution; (c) seed biopriming with microbial pellet; (d) microbial pellet with 5% PEG 6000 (e); microbial pellet with 10% PEG 6000; (f) microbial pellet with 15% PEG 6000; (g) microbial pellet with 20% PEG 6000; and (h) microbial pellet with 25% PEG 6000. Isolate SH-8 was grown in Luria–Bertani (LB) broth at 25–28°C for 4 days and was centrifuged at 3,000 × g. Thereafter, the obtained pellets were diluted with sterilized distilled water.

Each Petri dish was inoculated with 10^5^ colony-forming units (CFUs). Furthermore, in groups (d), (e), (f), (g), and (h), the Petri dishes were co-inoculated with PEG 6000 at a concentration of 5%, 10%, 15%, 20%, and 25%, respectively. For group (b), the isolate SH-8 was grown in LB broth and centrifuged at 3,000 × g for 5 min at 4°C, and the obtained supernatant (only culture broth/no pellet) was added to the Petri dish. After 8 days, the plants were harvested, and morphological parameters were noted. In the current study, PEG 6000 was selected to induce artificial osmotic stress because it modifies osmotic potential and induces water scarcity in a relatively controlled manner. PEG has a higher molecular weight than water, so it cannot cross the plant cell wall and is therefore used to rheostat the water potential in germination tests (Mille et al., 2005; Basha et al., 2015).

Two filter papers and an equal number of seeds (*n* = 10) were placed in each Petri dish, which was sealed with parafilm and then placed in a plant growth chamber at temperatures of 25−30°C (day) and 16−18°C (night) a humidity of 60% (day) and 80% (night). The germination potential (GP) of individual seeds was recorded every 24 h until no further germination occurred. After 8 days, the seedling length was measured. After completion of the experiment on day 8, the GP, GE, SVI, and germination rate index (GRI) were determined using the following equations (Al-Mudaris, 1998; Almaghrabi, 2012; Bhatt et al., 2015; Omidi and Mosavi, 2019):


(2) GP = no. of seeds germinated/total no. of seeds ×100

(3) SVI = [seedling length (cm) × germination percentage]

(4) GE = no. of germinated seeds on days 4 and 7/total no of seeds ×100, G4/10  ×100, G7/10 × 100

(5) GRI = ΣG/T

### 2.9 Seedling length

Three seedlings were randomly selected from each treatment group. The seedling length was measured from the growing meristematic tip to the point of attachment to the seed and was expressed in centimeters (cm), in accordance with the method described by Ghosh et al. (Ghosh et al., 2003)

### 2.10 Seedling weight

Three seedlings were randomly selected to measure the dry and fresh weights of the seedlings. Dry weight was measured after seedlings had been kept in a hot, dry oven for 1 day at a temperature of 82–84 ± 2°C and was expressed in milligrams (mg), in accordance with the method described by Yao et al. (Yao et al., 2012).

### 2.11 Bioassay on the bacterial isolates

SH-8 was chosen for this research. The isolated bacterial strains were screened based on their diverse PGP traits. The entire procedure followed that described in a previous publication by Kim et al. (Kim et al., 2020), with slight modifications. The bacterial isolates were also screened for the quantified production of abscisic acid through gas chromatography–mass spectrometry (GC-MS). The procedure followed was that described by Adebowale and Lawal (Adebowale and Lawal, 2003), with slight modifications. For sucrose quantification, 2.5* *mL of the SH-8 bacterial isolate culture was incubated for 3–5 days at 25–30°C and then centrifuged (at 5,000* *rpm for 15* *min). The product of centrifugation was filtered through a C18 Sep-Pak filter cartridge; thereafter, it was evaluated using the high-performance liquid chromatographic (HPLC) technique described by Kim et al. (Kim et al., 2020).

### 2.12 Oxidative stress tolerance

The oxidative stress tolerance of the SH-8 bacterial isolate was also tested. The entire procedure of testing for oxidative stress tolerance that followed was that described by Fan et al. and Rodríguez-Rojas et al. (Fan et al., 2015; Rodríguez-Rojas et al., 2020). To determine catalase activity, the Amplex Red Catalase Assay Kit (Invitrogen, Waltham, MA, USA) was used; for superoxide dismutase measurements, a SOD Assay Kit-WST (water-soluble tetrazolium) (Eugene, 1850 Millrace Dr #1, USA) was used; and the APX content was determined based on spectrophotometric absorbance at 290 nm.

### 2.13 Screening for exopolysaccharides

The Congo red agar assay was used to identify the presence of exopolysaccharides if produced by the bacterial isolate. Assay plates were prepared by mixing in LB broth (25 g/L), agar (2%), sucrose (5%), and Congo red (0.8 g/L). All chemicals were well mixed and then autoclaved. Microbes were allowed to grow on the assay plates for 5 days at 30–35°C. The observation of black colonies, visible against the red background, confirmed the presence of polysaccharides (Gandhi et al., 2017; Yang et al., 2022).

### 2.14 Scanning electron microscopy

All strains were allowed to grow on LB agar media plates. Thereafter, the agar surface was sliced into 5 × 2 × 5 mm (W × D × H) sections and placed in an Eppendorf tube, rinsed three times in 0.1 M sodium phosphate buffer, with a pH of 7.3, for 20 minutes, washed again in 2.5% glutaraldehyde solution, and finally incubated at 4°C for 2 hours, after which it was cleaned for 40 minutes with phosphate buffer solution. The samples were then connected with carbon tape and coated with Au–Pd after being dried off their moisture at a critical point. Following the addition of 100% isoamyl acetate, the samples were examined with an electron microscope (Hitachi S -3500N). The procedure of scanning electron microscopy (SEM) followed was that described by Shaffique et al. (Shaffique et al., 2022a).

### 2.15 Statistical analysis

MEGA X software (Version 7.222) was used to construct the phylogenic tree for molecular characterization. The randomized experimental designs were replicated five times. For graphical representation, GraphPad Prism software, version 6.0 (Dotmatics, San Diego, CA, USA), was used. The mean values were calculated using Duncan’s multiple range test (DMRT).

## 3 Results

### 3.1 Growth promotion assay results

The bacterial strain was assessed for the production of siderophores, IAA, exopolysaccharides, and phosphate solubilization potential to evaluate its growth-promoting traits. The results are given in **Table 1**.

**Table 1:** The plant growth-promoting (PGP) traits of the novel bacterial strain SH-8.

PGP traits	SH-8
Phosphate solubilization index	4.14 ± 0.30
Production of indole acetic acid	+++
Siderophore production	+++
Production of exopolysaccharides	+++

+++ indicate higher production of compnent, ± indicate standard deviation.

The results presented in **Table 1** show that SH-8 has significant phosphate-solubilizing activity (PSI 4.14 ± 0.30).

**Figure 1:**
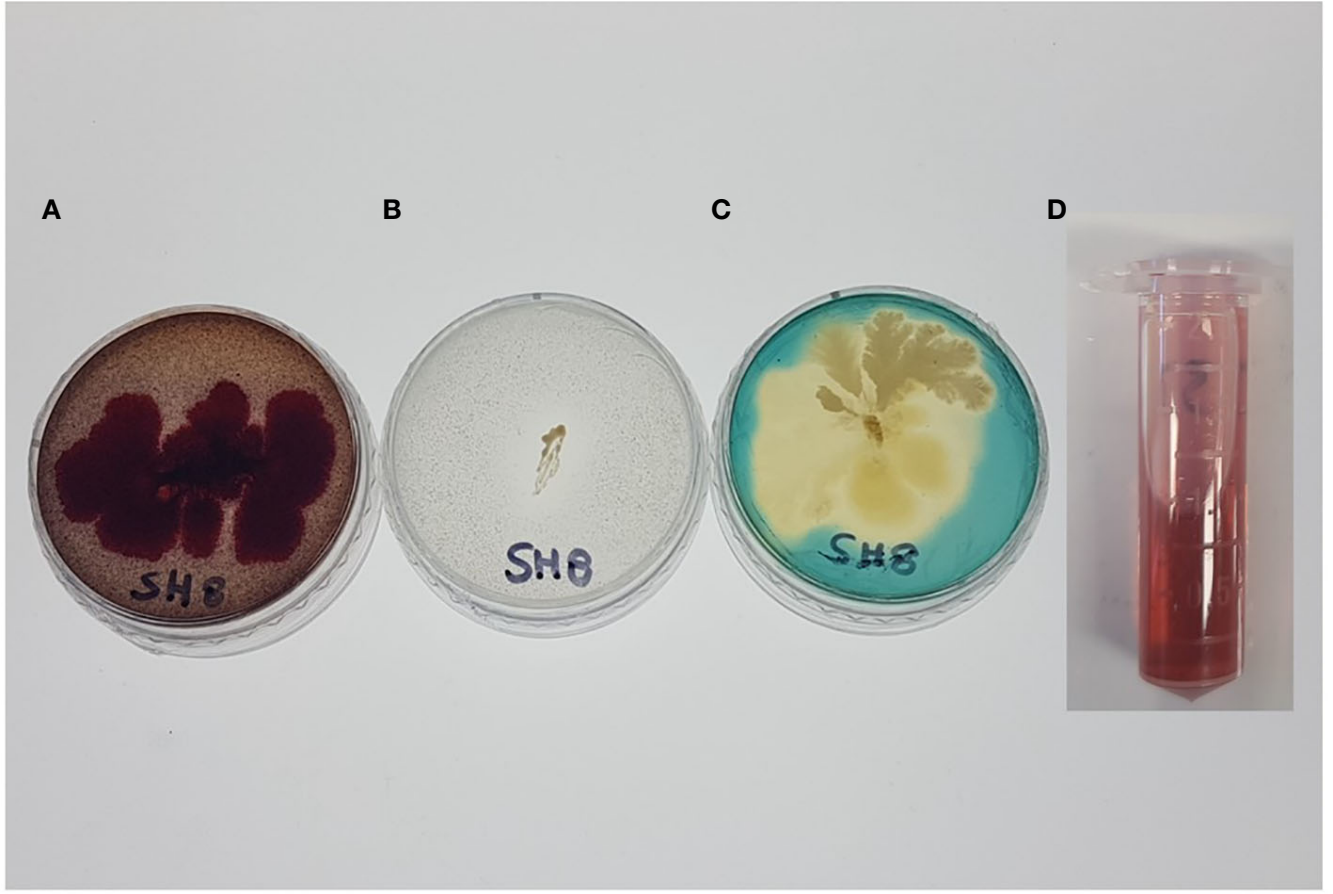
**(A)** Exopolysaccharide production, **(B)** phosphate solubilization, **(C)** siderophore production, and **(D)** indole acetic acid production by the novel bacterial isolate SH-8.

The results presented in **Table 2** show that SH-8 has significant phosphate-solubilizing activity. This bacterial isolate was also screened for the production of siderophores. The presence of transparent halos or the light-orange color around the isolate was considered indicative of positive siderophore production, as shown in **Figure 1**.

**Table 2:** Phosphate solubilization index (PSI) of the novel bacterial isolate SH-8.

Bacterial isolate	Colony diameter (cm)	Halo zone diameter (cm)	PSI
SH-8	1.68 ± 0.11	5.28 ± 0.25	4.14 ± 0.30

± indicate standard deviation.

### 3.2 Molecular identification results

**Figure 2:**
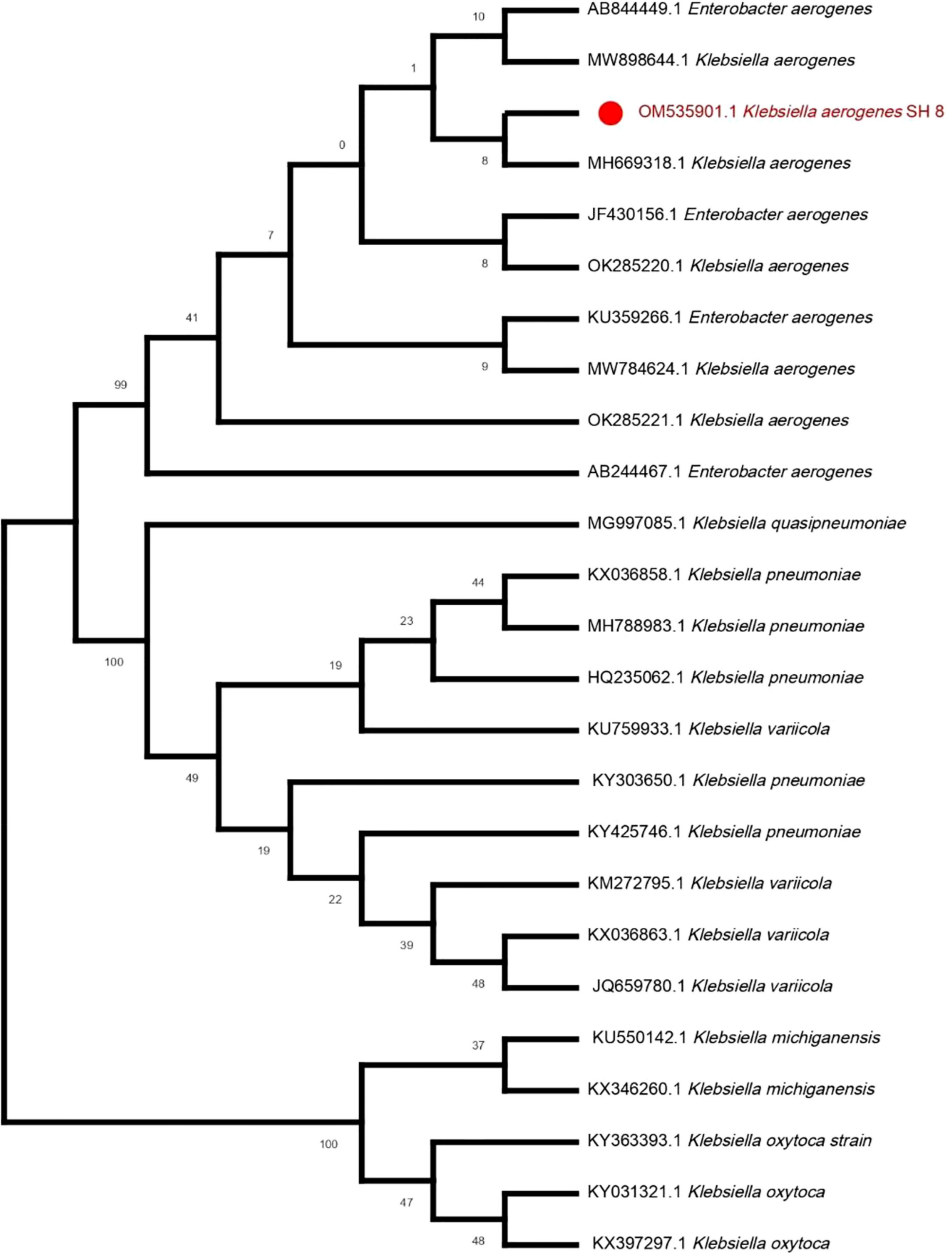
The phylogenetic tree of *Klebsiella aerogenes* SH-8 was constructed from 16S rRNA sequences using the ML method with the aid of the MEGA X program.

Owing to it having the best PGP characteristics, SH-8 was selected among all the isolates. The gene sequence of SH-8 was submitted to GenBank at NCBI for molecular identification and was assigned the unique gene accession number OM535901. Our analysis of SH-8 for molecular identification and phylogenetic analysis indicated that SH-8 had a sequence similarity of 99.93% with the *K. aerogenes* strain H217. The phylogenetic analyses also showed the same result, and SH-8 and *K. aerogenes* (MH669318) formed a clade using the ML method (**Figure 2**).

### 3.3 Abscisic acid and sucrose quantification results

**Figure 3:**
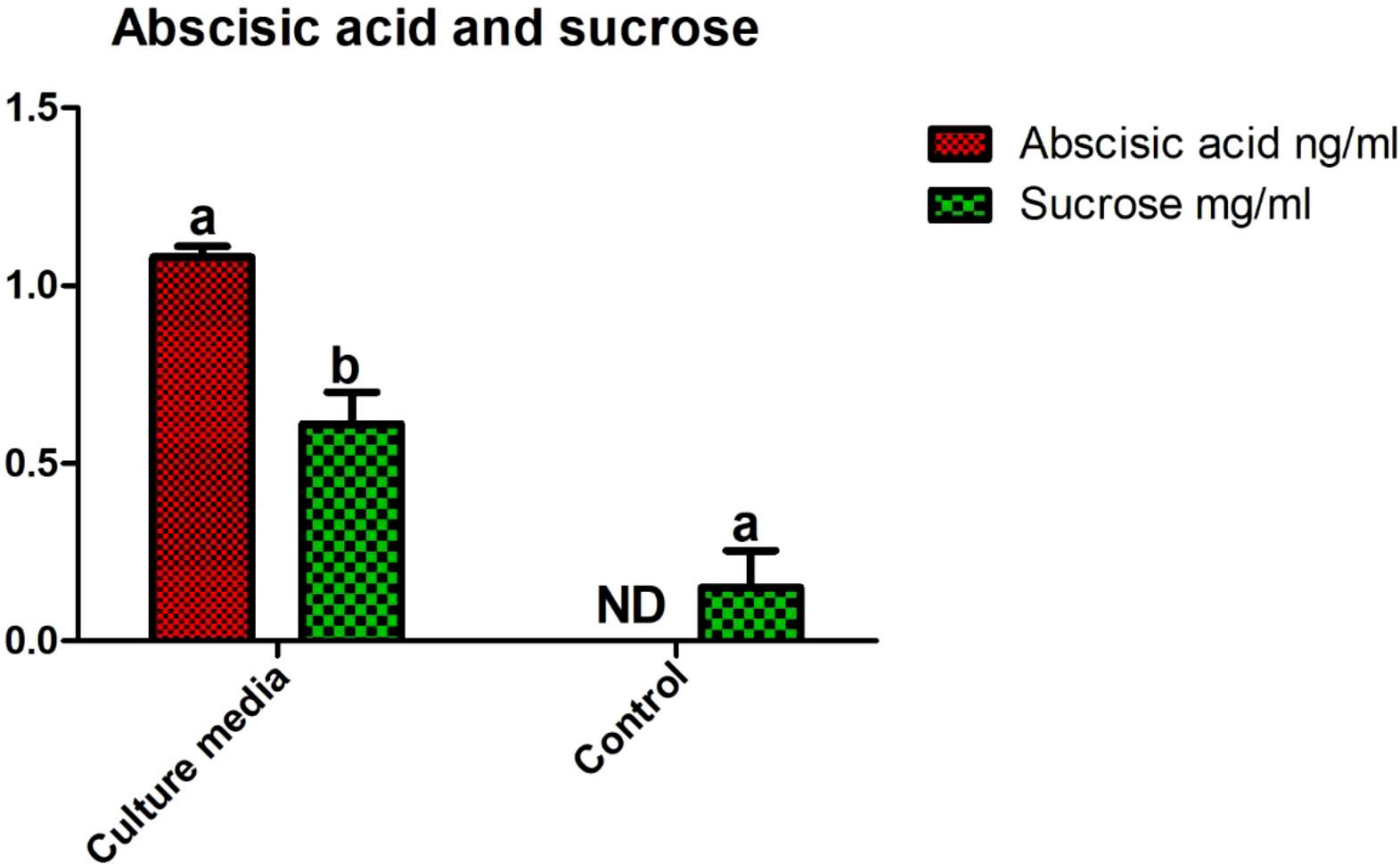
Abscisic acid and sucrose quantification in SH-8 cultured on Luria–Bertani broth. Each data point is the mean of five replicates. Error bars represent the standard error.

Abscisic acid (ABA) produced by the bacterial isolate was quantified using GC-MS. The results showed that a significant amount of ABA hormone (1.08 ± 0.05 ng/mL) was produced by the novel isolate bacterial strain SH-8. Sucrose quantification is important for estimating the formation of exopolysaccharides by the strain. The HPLC results showed that SH-8 produces significant amounts of sucrose (0.61 ± 0.13 mg/mL) as compared with the control group (only Lb media or non-incocluate with SH-8) (0.04 ± 0.004 mg/mL). LB media was taken as a control group. The results are shown in **Figure 3**.

### 3.4 Oxidative stress tolerance results

**Figure 4:**
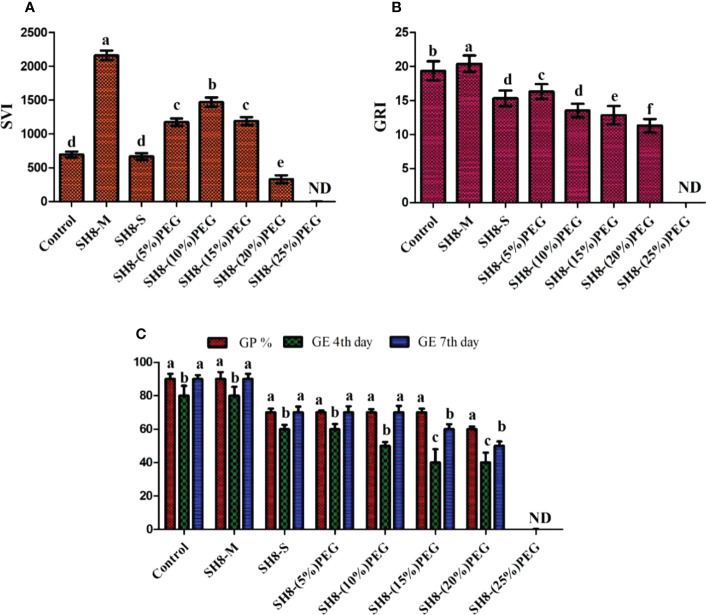
Production of significant amounts of superoxide dismutase **(A)**; catalase **(B)**, and ascorbic peroxidase **(C)** in the SH-8 culture broth media and cell pellet. Error bars represent the standard error.

The novel bacterial strain showed both a high tolerance to oxidative stress and significant growth on LB agar media plates containing up to 2 mM hydrogen peroxide. Similarly, the culture media of SH-8 showed considerably higher activity levels of catalase (CAT), superoxide dismutase (SOD), and ascorbic peroxidase (APX), representing its highly active antioxidant mechanisms. The results are shown in **Figure 4**.

### 3.5 Polyethylene glycol tolerance results

**Figure 5:**
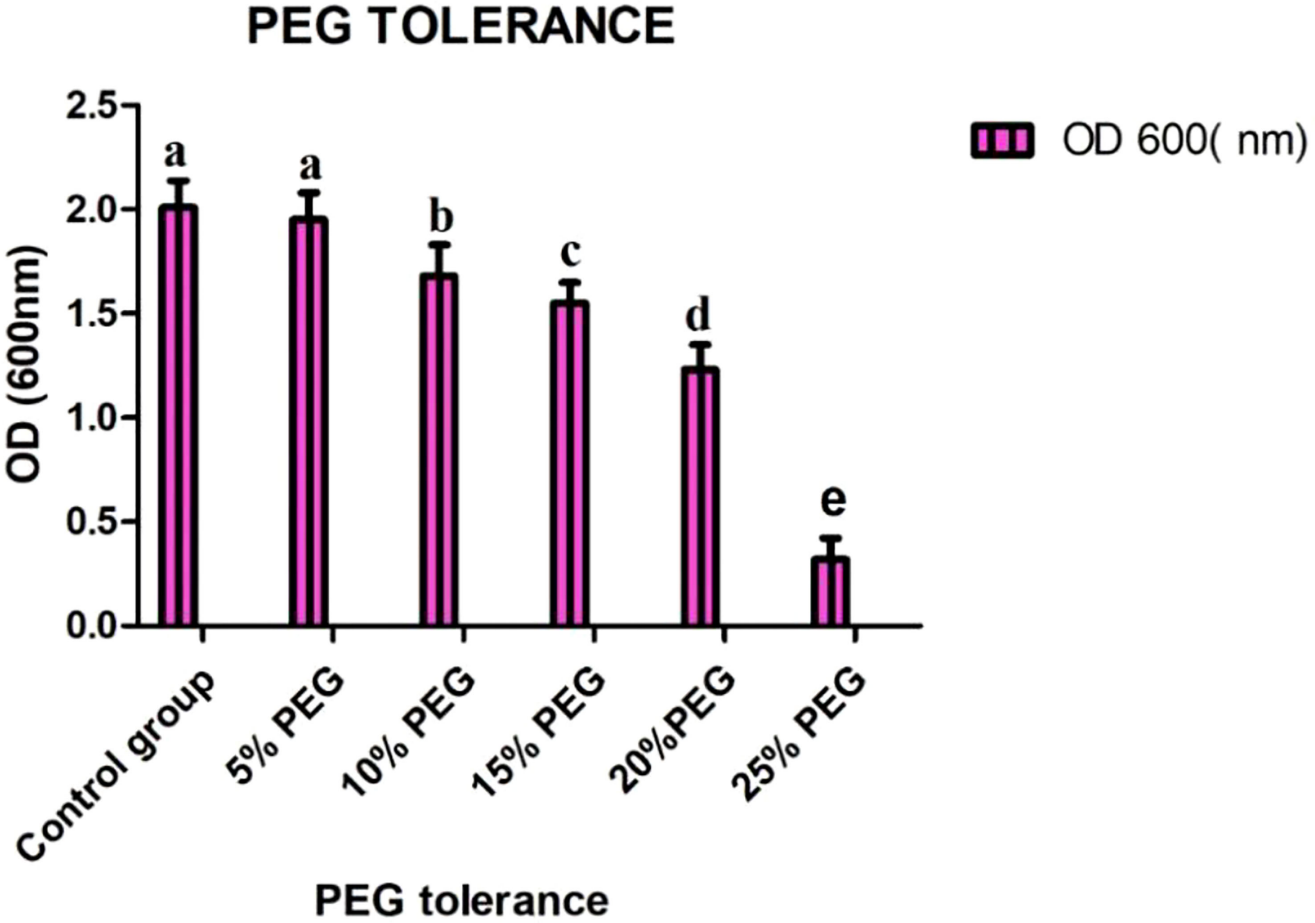
Growth of SH-8 with different concentrations of PEG 6000. Each data point is the mean of five replicates. The error bar represents the standard error. Different lowercase letters indicate significant difference in treatment.

The bioassay for SH-8 showed significant PGP traits, which were further screened with different concentrations of polyethylene glycol (i.e., 0%, 5%, 10%, 15%, 20%, and 25%). Among all isolates, SH-8 showed the highest PEG tolerance. The results are shown in **Figure 5**. Among all the strains, based on their high PEG tolerance, high PSI, significant production of ABA and sucrose, and considerable tolerance to oxidative stress, our team concluded the superiority of the SH-8 bacterial strain.

### 3.6 Results of *s*canning electron microscopy

**Figure 6:**
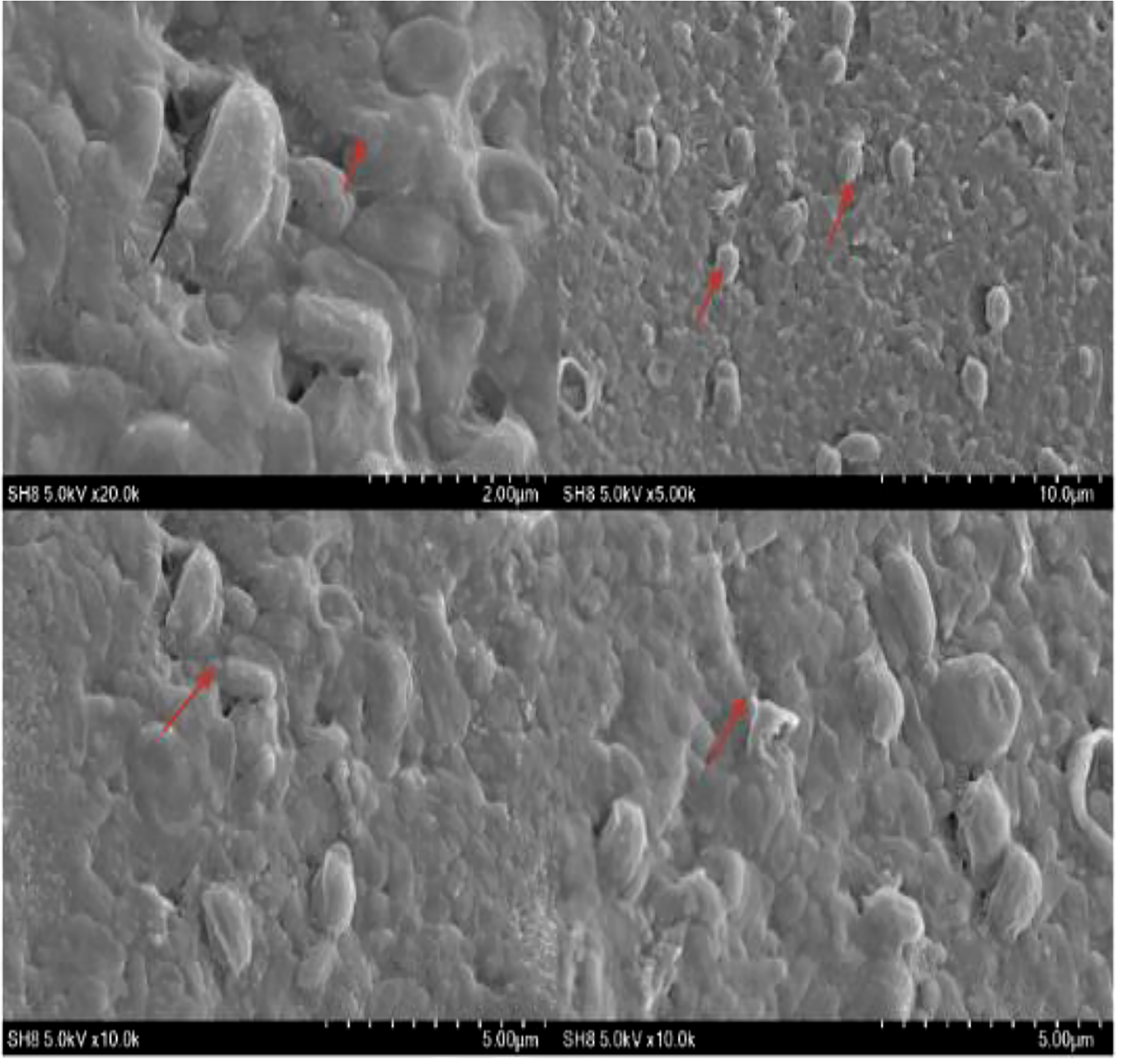
Scanning electron microscopy at a different resolution. The arrows indicate the presence of the biofilm and surface characteristics of bacterial isolate SH-8.

Scanning electron microscopy (SEM) revealed the presence of a bacterial biofilm and allowed observation of its surface properties. Our team recently reported the biochemical, adhesive, and biofilm characteristics of various novel bacterial strains, including SH-8 (Shaffique et al., 2022a). Here, we report the results from a preliminary study, including the results of SEM. These reveal the potential use of bacterial strains and their transduction ability to form adhesive biofilm. The SEM findings also confirm that the bacteria are embedded in an exopolysaccharide matrix, as shown in **Figure 6**.

### 3.7 Results of the seed biopriming assay

Wheat seeds were inoculated with the novel bacterial strain *via* a seed biopriming technique. Seed biopriming affects seedling length through the phenomenon of prime-omics (Adimoelja, 2000). Root and shoot elongation and dry and fresh biomass were higher in the SH-8–(10%) PEG group than in the control group. The germination potential was 70%, with a high SVI of 1,470. The germination energy was 50% on the fourth day, whereas it was 70% on the seventh day. The growth rate index was 12.4%, which was comparable to that of the control group. All the results of the germination potential assay are shown in **Figure 6**. A relatively greater root length, that is, 15 ± 0.21 cm, was recorded for SH-8–(10%) PEG-treated seedlings than for seeds bioprimed with other treatments. This could be because of the development of defense mechanisms in the SH-8-treated seeds, including increased antioxidant production and changes in osmotic processes. This mechanism originates from the primary memory, which can be established after secondary exposure, and is known as prime-omics (Khomari et al., 2018; Srivastava et al., 2021) (Aswathi et al., 2021). The germination percentage decreased linearly with an increase in the concentration of PEG. In the SH-8–(25%) PEG-treated group, there was a complete inhibition of germination to 0%. In addition, in seeds treated with SH-8–(20%) PEG, a germination potential of 60% was observed. In the SH-8-M (micro pellet)-treated group, the average root length and hypocotyl length were 17 ± 0.24 and 7 ± 0.28 cm, respectively; these lengths were greater than those for the SH-8-S-treated group, which were 5 ± 0.41 and 4.5 ± 0.84 cm, respectively, as shown in **Table 3**.

**Table 3:** Biomass and early seedling characteristics of the drought-treated seedling (root and shoot length grown for 8 days).

Treatment	Fresh biomass (mg)	Dry biomass (mg)	Root length (cm)	Shoot length (cm)
SH-8-M	28.25 ± 0.06 a	1.93 ± 0.08 a	17 ± 0.24 a	7 ± 0.28 a
SH-8-S (microbe solution)	22.66 ± 0.04 e	1.91 ± 0.05 b	5 ± 0.41 d	4.5 ± 0.84 d
SH-8−(5%) PEG 6000	26.52 ± 0.03 b	1.88 ± 0.07 c	15.8 ± 0.23 b	5.9 ± 0.52 c
SH-8−(10%) PEG 6000	25.37 ± 0.05 c	1.78 ± 0.07 d	10.8 ± 0.22 c	5.7 ± 0.55 c
SH-8−(15%) PEG 6000	20.66 ± 0.03 f	1.25 ± 0.03 g	10.1 ± 0.8 c	5.9 ± 0.62 c
SH-8−(20%) PEG 6000	19.99 ± 0.09 g	1.33 ± 0.06 f	3.5 ± 0.9 e	2 ± 0.22 e
SH-8−(25%) PEG 6000	0 ± 0.0 g, h	0 ± 0.0 h	0 ± 0.0 f	0 ± 0.00 f
Control	24.33 ± 0.32 d	1.52 ± 0.18 e	15.2 ± 0.7 b	6.2 ± 0.12 b

Values are presented as the mean of six replicates ± standard deviation (SD). Values followed by different lower-case letters are significantly different at a p-value< 0.05 in all treatment groups.

The results indicated that pellet treatment, rather than bacterial solution treatment, was the preeminent treatment. The nutrient broth in which inoculum is prepared, however, is sometimes toxic to the plants, which may create fallacies in exact data generation. Moreover, using a pellet only can help distinguish the particular effect and generate precise data; hence, we used the bacterial pellet (Penrose and Glick, 2003; Abou-Shanab et al., 2008). SH-8–(15%) PEG, when applied as a treatment, resulted in an increase in the root length to 10 ± 0.22 cm and in hypocotyl length to 7 ± 0.37 cm, which were larger than the increases observed in the control group. The fresh and dry biomasses of the seedlings were measured, and the results are presented in **Table 3**. The fresh and dry biomass of seedlings treated with 10% PEG was 25.37 ± 1.55 mg and 1.78 ± 0.07 mg, respectively, which were significantly higher than those in the control group and comparable to those in the SH-8–(15%) PEG group.

## 4 Discussion

Wheat is an important cereal crop that is cultivated all around the world. It is one of the top three most-grown cereal crops, along with maize and rice, because of its high nutritional value and is a major source of carbohydrates and energy. It also contains folate, proteins, fibers, calcium, and magnesium (Shewry, 2009; Wa Thiong'o, 2012). The productivity rate of whole wheat is lower than that of cereal crops, despite it having the largest total harvested area and being most in demand globally. Wheat production has been greatly affected by environmental changes, the increase in demand brought about by the population increase, and the fact that 35%–45% of the world’s land area is semiarid and is under varying degrees of ecological stress. Environmental (biotic and abiotic) stresses are the foremost cause of agronomic productivity loss (Cherry, 2013; Mareri et al., 2022).

Anthropogenic activities have exacerbated the negative impacts of climate change and have an adverse effect on ecological systems. Due to climate change and a shifting regime, plants encounter various environmental stresses that restrict their productivity. It is estimated that 50% of crop production is lost due to abiotic stress, such as drought stress (Jenks and Hasegawa, 2008; Pereira, 2016). The agriculture industry faces two main challenges: one concerns the impact of abiotic stress on plant growth, and the other is meeting the needs of an increasingly international population. It is estimated that, by 2050, the global population will have increased to 9.8 billion. To enhance agriculture production, sustainable agronomic techniques should be implemented. The capacity of existing techniques to enhance crop productivity under climate change, however, is limited (Asenso-Okyere and Jemaneh, 2012; Wang et al., 2015). Drought stress drastically reduces crop production and negatively affects crop quality. However, the effects of drought stress can be mitigated by various new, promising approaches, including approaches at the seed level (Salehi-Lisar and Bakhshayeshan-Agdam, 2016; Kaur and Asthir, 2017).

Drought is an imperative environmental stress that cannot be avoided because of the immobile nature of plants and the presence of semiarid and arid areas on our planet (Ozturk et al., 2021). Plant–microbial interactions play a very important role in combating drought stress. Plant–microbe interactions play an important role in enhancing drought stress tolerance *via* metabolic reprogramming. When microorganisms interact with plants, microbe produce certain metabolites and phytohormones that maintain the osmoregulation of the plant cell (Vaishnav et al.; Singh et al., 2004). Seed biopriming is a new approach to inoculating seeds with microbes to promote the plant–microbe interaction (Zaher et al., 2022). Biopriming is emerging as an economically feasible and environmentally friendly technique that can be used to improve plant tolerance to abiotic stresses, and interest in this technique continues to grow because of its sustainable role in agriculture (Reddy, 2012; Mahmood et al., 2016). The improvement in plant drought tolerance and germination can be attributed to the growth-promoting effect of biopriming (Bisen et al., 2015). In the present study, the PGP activity of the isolated bacterial strain SH-8 was analyzed. The results showed that because of its versatile growth-promoting characteristics, such as phosphate solubilization and the production of siderophores, exopolysaccharides, and phytohormones, this isolate is a PGP agent and not a pathogen. These growth-promoting characteristics are discussed in detail below.

Phosphorus is a necessary macronutrient, required for plant growth at all stages, from seedlings to vegetation. The results showed that SH-8 hydrolyzes insoluble phosphorus to its soluble form, thereby helping to increase its uptake and further assimilation and decreasing plant dependence on phosphorus from the external environment (Roslan et al., 2020; Saheed and Ikhajiagbe, 2021). It was found that SH-8 has a positive phosphorus solubilization potential, with a PSI of 4.14 ± 0.30, increasing drought tolerance by up to 15%. Bacterial strains can solubilize phosphorus owing to their ability to reduce the pH of the surrounding area *via* the production of organic acids. These organic acids function as anion exchangers and convert the insoluble form of phosphorus into the soluble form (Sundara et al., 2002; Khan et al., 2009; Wan et al., 2020). Thus, the use of phosphate-solubilizing bacteria has great potential for sustainable agronomy (Elkoca et al., 2007; Kour et al., 2019b).

We also set out to determine if SH-8 produces IAA. The IAA-producing potential of SH-8 was measured, and the results showed that the isolate does indeed produce this compound. IAA plays a crucial role in seed germination. It is an important phytohormone that regulates biological processes and enhances the stress tolerance of plants. It also regulates cell proliferation and differentiation (Sandberg and Ernstsen, 1987; Shuai et al., 2016). The ability of SH-8 to produce exopolysaccharides was also investigated, and the results confirmed that it does produce exopolysaccharides, which are important for bacterial attachment to the plant surface, and the subsequent formation of biofilm (Naseem and Bano, 2014; Silambarasan et al., 2019). The ability of this new bacterial strain to produce siderophores was also investigated, and the results showed that it has the potential to produce siderophores, whose activity is crucial to ensuring that plants absorb an adequate amount of iron. SH-8 application restored the nutritional status of plants and could be applied as a biostimulant agent for plant production under various stresses (Arora et al., 2001; Deshwal et al., 2003).

Biopriming causes an increase in ATP synthesis, which promotes selective absorption and defense mechanisms in plants (Chakraborti et al., 2021). Seed biopriming also maintains cytomembrane stability and chlorophyll synthesis (Chandra Nayaka et al., 2010; Choudhary and Varma, 2016). Drought tolerance may be enhanced by the selective uptake mechanism promoted by seed biopriming, which restricts the absorption of PEG in plant cells.

Drought stress causes the accumulation of reactive oxygen species (ROS), which contribute to protein degradation and cellular toxicity (Kaur and Asthir, 2017; Qi et al., 2018). Biopriming enhances the development of the antioxidant system consisting of APX, SOD, and CAT, which scavenges free radicals and reduces their cellular toxicity (Yadav et al., 2012; Panuccio et al., 2018). The bioassay of SH-8 showed that the levels of APX, SOD, and CAT were significant. Drought tolerance may also be enhanced by the redox signaling defense mechanism induced by the SH-8 bacterial isolate. It acts as a defense strategy in the mitigation of abiotic stress (Fujita and Hasanuzzaman, 2022; Hasanuzzaman and Fujita, 2022).

**Figure 7:**
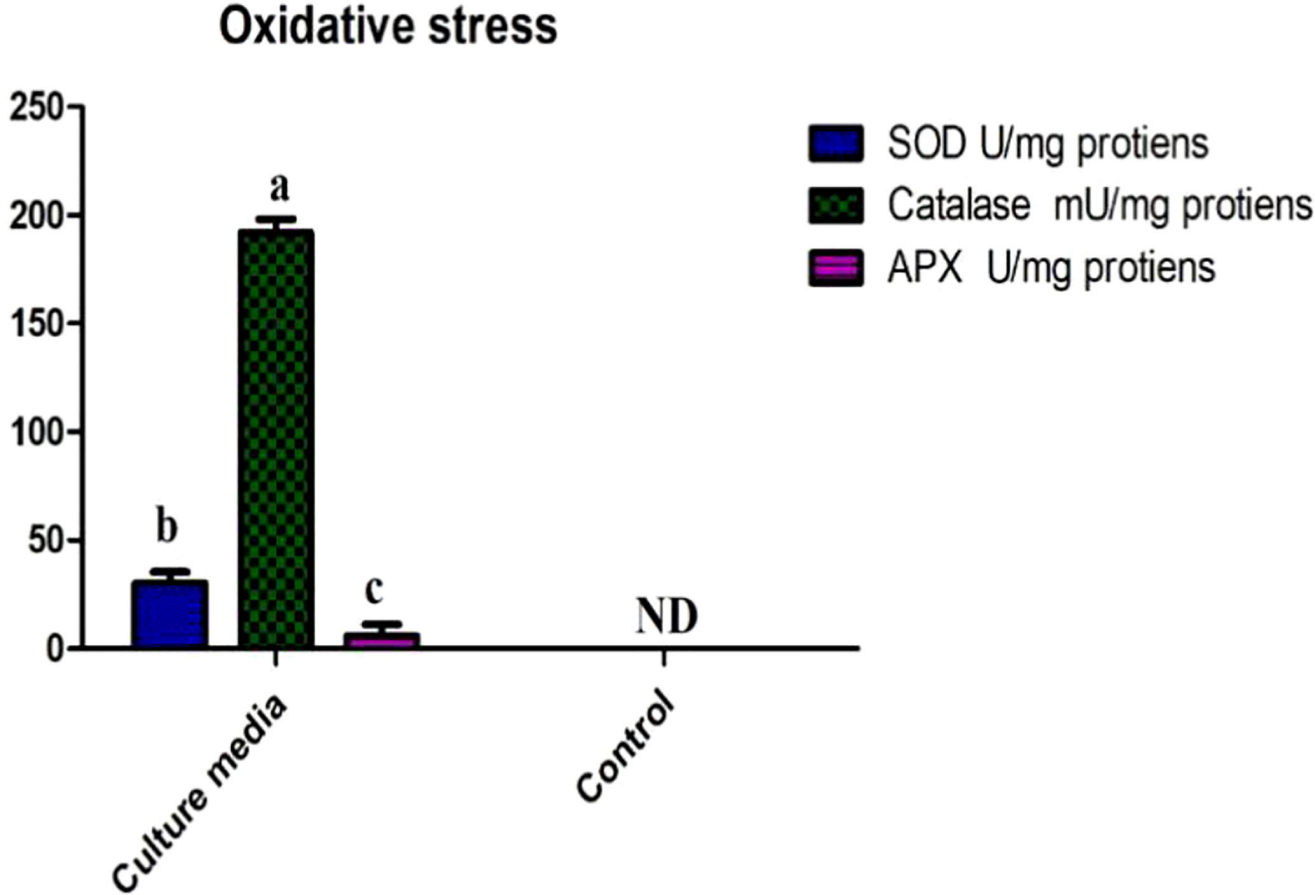
Effect of various concentrations of PEG 6000 and biopriming with SH-8 on the seed vigor index, germination rate index, germination energy, and germination potential of wheat seeds. PEG, polyethylene glycol. Different lowercase letters indicate significant difference in treatment.

**Figure 8:**
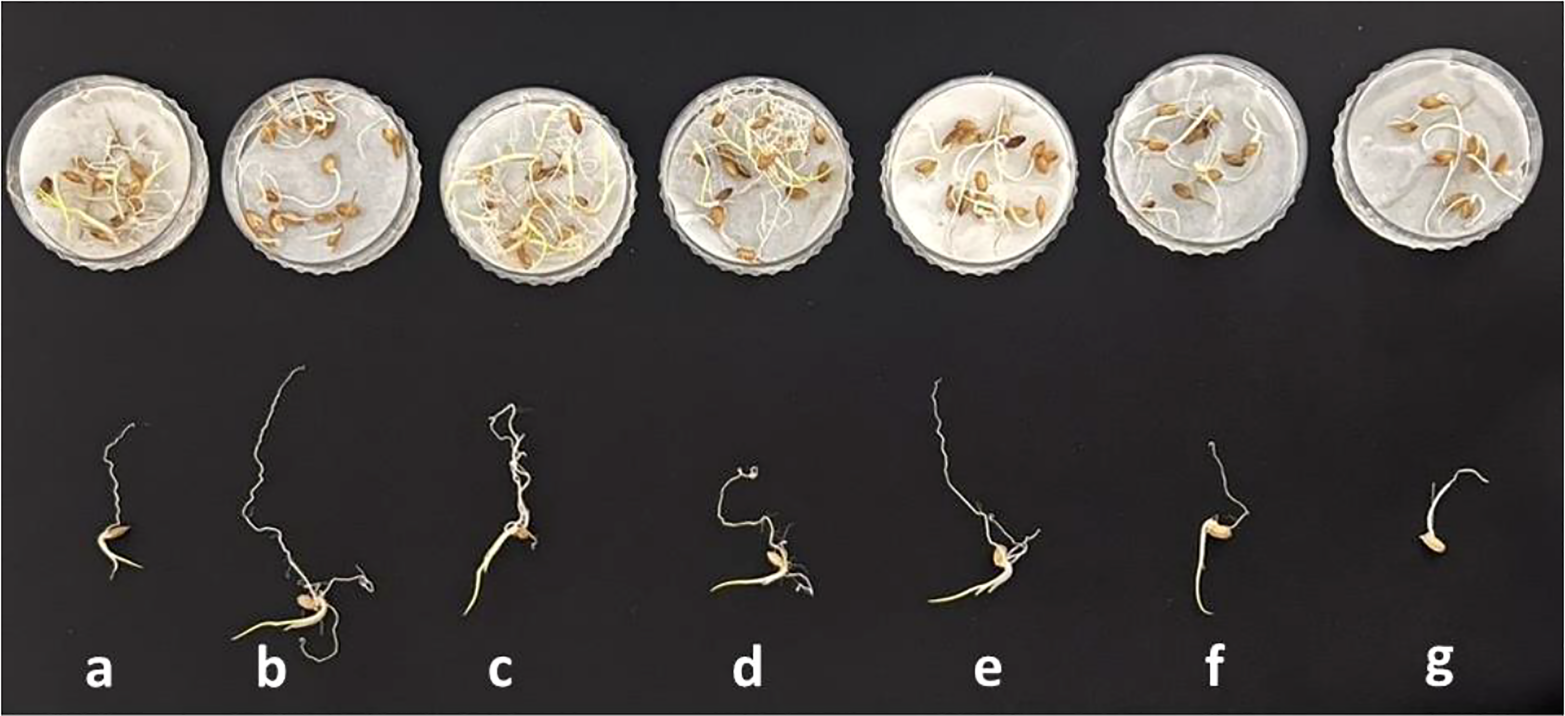
*In vitro* seed biopriming with different concentrations of the polyethylene glycol: **(A)** normal control; **(B)** microbial pellet; **(C)** microbial pellet + 5% PEG **(D)** microbial pellet + 10% PEG; **(E)** microbial pellet + 15% PEG; **(F)** microbial pellet + 20% PEG; and **(G)** microbial solution. PEG, polyethylene glycol.

With increasing stress levels, the germination potential progressively decreases. The bacterial isolate SH-8 can increase tolerance to stress induced by the presence of PEG only at concentrations up to 20%. After greater stress levels are induced by higher PEG concentrations, germination is totally inhibited. As shown in **Figure 7**, germination did not occur in plants inoculated with SH-8 and 25% PEG; in this case, total inhibition of germination was observed. Therefore, as shown in **Figures 7**, **8**, it can be concluded that SH-8 can tolerate stress induced by PEG at concentrations up to 20%.

Phytohormones also play a significant role in stress tolerance. Various studies have suggested that ABA, by promoting stomatal closure and water retention, is important in stress mitigation (Per et al., 2017; Ullah et al., 2018; Jochum et al., 2019; Jogawat et al., 2021). The results in **Figure 3** show that plants bioprimed with SH-8 contained significant levels of ABA, which is a stress hormone that is an antagonist of ethylene and maintains the vigor index by lowering the level of ethylene. ABA is an endogenous phytohormone that is released in large quantities immediately after stress exposure. It modulates stress *via* the activation of NADPH oxidases, sucrose metabolism, and the regulation of proteostasis. SH-8 was also screened for IAA production. The results suggested that SH-8 contains a significant amount of IAA. IAA also modulates drought stress *via* its regulation of certain genes and provides cellular stability (Li et al., 2018).

In the present study, the effects of stress were mitigated by 20% by biopriming seedlings with SH-8 while maintaining their characteristics and sustaining the cytomembrane. However, it should be noted that this is an *in vitro* study conducted on a small scale. Further molecular-level studies should be conducted to determine the mechanism of action. The optimum PEG stress level was 10%, which initiated seedling growth in wheat plants without causing any gross abnormalities. The identification of the most effective stress-inducing dose of PEG is important for plant recovery from osmotic stress. Seed biopriming, a defense mechanism that protects the seeds when they are competing in unfavorable conditions, improves the germination capacity of plants. In addition, seed biopriming improves the energy status of the mitochondria and enhances protein synthesis, which also leads to improved germination (Sukanya et al., 2018; Samal et al., 2020; Negi and Bharat, 2021). Owing to the significance of the seed vigor index, germination potential, and growth rate index, PEG concentrations of over 10% did not improve plant drought tolerance *in vitro*. SH-8 will serve as a reliable biopriming treatment option for induced osmotic stress of 15%. This optimum stress level, together with seed biopriming, is the best option for developing drought tolerance in plants and could be used to improve wheat growth programs in the future.

In this study, we isolated a *Klebsiella*-like bacterial strain, SH-8, that produces the phytohormone ABA, which was found to produce 1.08 ± 0.05 ng/mL (**Figure 4**). This relatively high abundance of ABA is very unusual, being higher than the abundances found in other bacterial strains (Shahzad et al., 2017; Khan et al., 2019; Kim et al., 2020). Previous studies have reported that hormonal pretreatment may encourage stress tolerance in growing seeds by increasing the number of required enzymes such as alpha-amylase and maintaining ionic and hormonal balance (Farooq et al., 2009). This suggests that successful germination depends on the plant embryo’s ability to develop its metabolic activity. However, several environmental factors and plant hormones affect the metabolism and different hormonal signaling pathways within plants (Holdsworth et al., 2008). For this reason, it is suggested that counterbalancing the levels of ABA and gentisic acid helps plants regulate seed germination under abiotic stress (Vishal and Kumar, 2018). We have also found that this bacterial strain produces higher levels of sucrose, that is, 0.61 ± 0.13 mg/mL (**Figure 3**), than a closely related strain of the bacterium *Klebsiella* (Le Rudulier et al., 1982; Khan et al., 2014; Asaf et al., 2017). This study also introduces a novel method for the enhancing drought tolerance in wheat subjected to oxidative stress as well as an improved method of measuring germination metrics and early seedling characteristics. Indeed, we found that strain SH-8 also produces high levels of siderophores, exopolysaccharides, and IAA. A possible interpretation of these results is that these factors promote plant growth and germination under osmotic stress.

## 5 Conclusions

The novel rhizosphere strain SH-8 can improve plant drought tolerance and exhibits different PGP traits, such as oxidative stress tolerance, the production of IAA and exopolysaccharides, phosphorus solubilization, and PEG tolerance. This novel bacterial strain was also shown to significantly increase plant biomass, and its inoculation improved plant germination metrics. SH-8 promoted the production of plant endogenous phytohormones and antioxidants (i.e., SOD, APX, and CAT), as well as drought stress regulators, minimizing the effects of ROS. Therefore, SH-8 can be used to improve drought tolerance and as an eco-friendly biostimulant for sustainable agronomy in drought-affected areas of the world.

## 6 Future perspectives

The present study indicates that SH-8 has great potential to enhance plant germination metrics under drought stress. However, although considerable evidence has been accumulated regarding the positive effect of SH-8 on germination and early seedling characteristics under drought stress, more research is required before this strategy becomes the mainstay of agricultural practice. Further research is also needed to understand the molecular mechanisms of stress tolerance on a larger scale. There is a growing awareness among researchers of the need to isolate more effective bacterial strains. Overall, it is anticipated that continued research on seed biopriming using beneficial stress-mediated strains in plants will result in this technology being used as a means of sustainable management in agriculture in the near future.

## Data availability statement

The original contributions presented in the study are included in the article/supplementary material. Further inquiries can be directed to the corresponding authors.

## Author contributions

SS conceptualized, wrote the draft, and did the experiment as part of her dissertation. MI, MAK, and S-MK assisted in paper revision and experimentation. While W-CK and SA did critical review, and proofreading. I-JL supervised, funded and validated results and final draft.
